# A Review of Chronic Comorbidities in People Living With HIV in Peru

**DOI:** 10.1177/23259582261470497

**Published:** 2026-07-17

**Authors:** Marleny Nolasco, Evelyn Hsieh, Patricia J. Garcia, Monica M. Diaz

**Affiliations:** 1 Department of Neurology, 6797 University of North Carolina at Chapel Hill School of Medicine, Chapel Hill, NC, USA; 2 Department of Medicine, Yale School of Medicine, New Haven, CT, USA; 3 School of Public Health, Universidad Peruana Cayetano Heredia, Lima, Peru

**Keywords:** HIV, chronic comorbidities, chronic illness, aging, Peru, Latin America

## Abstract

**Background:**

Advances in antiretroviral therapy (ART) have extended the life expectancy of people living with HIV (PWH) in Peru, shifting the healthcare focus toward managing chronic non-communicable comorbidities (NCDs). Yet data on NCD burden among aging PWH in Peru are sparse.

**Methods:**

We conducted a scoping review of peer-reviewed studies from PubMed and LILACS, in English or Spanish, that assessed non-AIDS comorbidities in Peruvian adults with HIV.

**Results:**

We identified 23 studies, all from Lima, mostly cross-sectional or retrospective, involving predominantly male ART patients. Commonly investigated comorbidities included neurocognitive impairment, mental health disorders (especially depression), cardiovascular disease, and metabolic syndrome; bone disease and non-AIDS malignancies were less studied.

**Conclusions:**

Findings suggest frequent cognitive, metabolic, and cardiovascular issues among PWH. Yet critical gaps remain: little is known about frailty, disability, or long-term outcomes. Expanded screening, longitudinal studies, and integrated care models are needed to improve long-term health in aging PWH in Peru.

## Plain Language Summary

**Background:**

Due to better HIV treatment, people with HIV in Peru are living much longer. As a result, their medical care now needs to focus more on long-term health issues, not just infections related to HIV, but other chronic diseases. However, there is little information about how common these chronic diseases are among older people with HIV in Peru.

**What we did:**

We looked for published studies (in English or Spanish) that reported on long-term health problems, not AIDS-related, in adults with HIV living in Peru.

**What we Found:**

We found 23 studies, all from the capital city, Lima. Most studies looked at men on HIV treatment, and were based on single-timepoint or past record data. The most common health problems reported were memory and thinking problems, depression and other mental-health issues, heart disease, and conditions linked to metabolism. There was less research on bone problems or non-AIDS cancers.

**What This Means:**

People with HIV in Peru are often dealing with health issues beyond HIV, especially problems affecting thinking, mood, heart health, and metabolism. But we still don’t know much about other serious concerns like frailty, disability, or how these issues change over time. We need better screening, long-term studies, and health care plans that include all these needs for older people living with HIV in Peru.

## Introduction

Low- and middle-income countries (LMICs) continue to confront the HIV epidemic, exacerbated by socioeconomic and healthcare infrastructure challenges that limit the development of comprehensive HIV prevention and treatment strategies.^1^ Since the widespread implementation of antiretroviral treatment (ART) in 2004, over 19.5 million people living with HIV (PWH) in LMICs have gained access to ART, including approximately 2.24 million individuals from Latin America and the Caribbean (LAC).^2^ While HIV prevalence in the Caribbean has shown a declining trend, the broader LAC region has achieved stable prevalence rates averaging 0.4%.^3^

As individuals live longer on ART, the focus has shifted toward the long-term, chronic complications of HIV and ART, particularly the increasing prevalence of aging-related comorbidities associated with long-term HIV infection and ART exposure.^4-6^ Non-communicable diseases (NCDs), such as cardiovascular disease (CVD), bone and musculoskeletal (MSK) disorders, malignancies, neurocognitive impairment (NCI), and mental health conditions have become significant health challenges among aging PWH (4-6). These conditions contribute to a growing burden of disease in PWH with implications for healthcare systems and patient outcomes.^4,7^

HIV continues to pose a significant public health challenge in Peru, where an estimated 110,000 adults live with HIV as of 2024.^8^ Peru has made significant progress in HIV control and treatment. However, the intersection of HIV with chronic comorbidities remains underexplored in Peru, as in many LAC countries. Aging PWH in Peru are increasingly experiencing the dual burden of lifelong ART exposure and the comorbidity of NCDs, similar to regional and global trends.^4,6^ Despite evidence of these challenges, data specific to Peru on the prevalence, risk factors, and outcomes associated with HIV-related NCDs are limited.

This review aims to address this critical gap by synthesizing available evidence on chronic comorbidities of HIV in Peru, with a focus on the burden of NCDs in aging PWH. Understanding the prevalence and impact of these comorbidities in the Peruvian context is critical to developing comprehensive public health strategies and national policies that address the aging of this population.

## Methods

**Figure 1.**
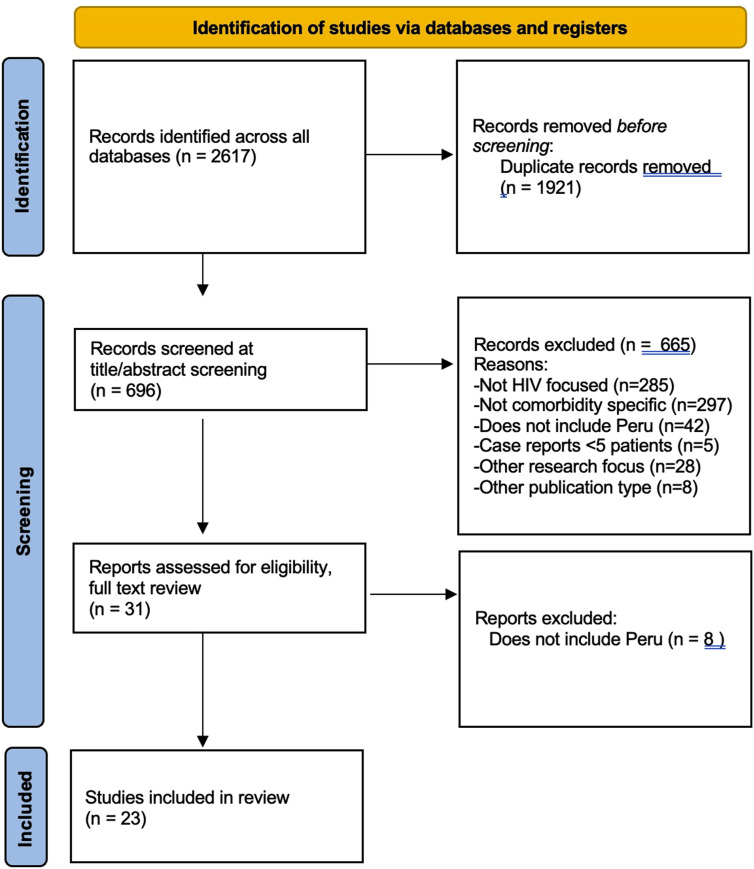
PRISMA Diagram demonstrating Studies included in the Review

We conducted a scoping review of existing published literature. We followed the guidelines outlined in the Preferred Reporting Items for and Meta-Analyses – Scoping Reviews (PRISMA-ScR) statement. We did not register the protocol prior to completing our search (Figure 1).

### Data Source and Searches

We searched two databases, PubMed and LILACS, to identify studies addressing HIV-associated chronic comorbidities in Peru. These databases were selected to capture both internationally indexed biomedical literature (PubMed) and regional publications from Latin America and the Caribbean that may not be indexed in global databases (LILACS). This combination has been commonly used in reviews of health research in Latin America to ensure inclusion of both global and regional sources. We searched PubMed and LILACS from database inception through December 2024 without applying language or publication date restrictions during the search process. However, for study inclusion, only full-text articles published in English or Spanish were considered eligible. Peru, “HIV”/“VIH,” and a range of keywords and controlled vocabulary terms related to comorbid conditions. The search strategy included combinations of “HIV” and “Peru” with the following comorbidity-related keywords: bone disease, non-AIDS-related malignancies, cancer, cardiovascular disease, cognitive impairment, comorbidities, cognition, depression, diabetes, dyslipidemia, hypertension, mental health, metabolic syndrome, metabolic disease, neurocognitive impairment, and sarcopenia. The complete, reproducible search strategy for all databases is available in the supplementary files.

While all searches must have included Peru and HIV concepts, the search results could have included any of the comorbidity terms listed in Table 1. Cproceedings and abstracts were filter out from the search.

**Table 1.** Search Term Combinations in English and Spanish (All Searches Included Peru and HIV)

Search query (English)	Search query (Spanish)
1. “HIV” OR “human immunodeficiency virus”	1. “VIH” OR “virus de la inmunodeficiencia humana”
2. “bone disease” OR “sarcopenia” OR “osteoporosis”	2. “enfermedad ósea” OR “sarcopenia” OR “osteoporosis”
3. “non-AIDS-related malignancies” OR “cancer”	3. “neoplasias no relacionadas con el SIDA” OR “cáncer”
4. “cardiovascular disease” OR “diabetes” OR “dyslipidemia” OR “hypertension” OR “metabolic syndrome” OR “metabolic disease”	4. “enfermedad cardiovascular” OR “diabetes” OR “dislipidemia” OR “hipertensión” OR “síndrome metabólico” OR “enfermedad metabólica”
5. “cognitive impairment” OR “cognition” OR “neurocognitive impairment”	5. “deterioro cognitivo” OR “cognición” OR “deterioro neurocognitivo”
6. “comorbidities”	6. “comorbilidades”
7. “depression” OR “mental health” OR “anxiety”	7. “depresión” OR “salud mental” OR “ansiedad”
8. Peru OR Peruvian OR Peruvians	8. Perú OR peruano OR peruanos
9. #1 AND (#2 OR #3 OR #4 OR #5 OR #6 OR #7) AND #8	9. #1 AND (#2 OR #3 OR #4 OR #5 OR #6 OR #7) AND #8

### Study Selection

Two independent screeners (MN and MMD) performed a title/abstract review, and MMD resolved any conflicts between two reviewers. All studies that met screening inclusion criteria were then reviewed in full text by two reviewers (MN and MMD), with MMD to resolve conflicts between two reviewers. Studies were eligible for inclusion if they met the following criteria: (i) peer-reviewed publications; (ii) observational cohort studies, case series with more than five participants, or clinical trials; (iii) conducted in Peru or included Peruvian participants in multi-country analyses; and (iv) focused on at least one HIV-associated chronic comorbidity. We excluded studies that were (i) not in English or Spanish; (ii) case reports, conference abstracts, opinion/commentary articles, or systematic reviews; (iii) studies that did not include Peruvian populations living with HIV; or (iv) did not mention one of the comorbidities of interest (Table 1).

### Data Extraction and Quality Assessment

Two reviewers extracted data independently (MN and MMD). The data was compared, and any conflicts were resolved by reviewer MMD. The data that were extracted from each eligible paper included study characteristics, sample size, HIV characteristics (i.e., recent absolute CD4 count, duration of HIV infection, HIV plasma viral load), and reported prevalence of the condition (if the prevalence was reported; prevalence was not required as an inclusion criteria). The JBI Critical Appraisal Checklist for Prevalence Studies was applied primarily to observational studies reporting prevalence estimates by the two reviewers (MN and MMD). Because included studies varied substantially in design and methodology, results of the appraisal were interpreted descriptively and were not used to determine study eligibility. We did not record the results of each individual item of the checklist for each study, but studies were reviewed to provide general context regarding methodological quality; appraisal findings were interpreted descriptively.

## Results

A total of 23 articles met the inclusion criteria and were reviewed for this study. All were published between 2007 and 2024, with a noticeable increase in publications over the past decade. Nearly half of the articles (11 out of 23) were published from 2020 onward. Of the 23 articles, 19 were strictly based in Peru,^9-26^ while 4 were international studies that included data from Peru.^27-30^ All of the studies included patients just from the capital city of Peru, Lima. Most studies (n = 18) were published in English. The majority were cross-sectional studies (15), five of these which were retrospective. Detailed characteristics of the included studies, including study design, sample size, participant characteristics, and primary findings, are presented in Table 2.

**Table 2.** All Studies Reporting HIV Comorbidities in a Peruvian Population

Title	Author	Comorbidity	Type of study	N	Mean age	Sex (% female)	Mean CD4	Study aims
Cognitive impairment and antiretroviral treatment in a Peruvian population of patients with human immunodeficiency virus	Guevara-Silva; 2014	NCI	Prospective Longitudinal study; Lima, Peru	19	33.5	26	Pre HAART: 142	The objective of the present study is to determine the association between cognitive impairment and HAART in HIV-infected patients from Hospital Regional de Huacho.
							Post HAART: 283	
Cognitive profile in human immunodeficiency virus-infected neurologically asymptomatic patients	Guevara-Silva; 2013	NCI	Prospective, Cross-sectional study; Lima, Peru	21	33	23.8	147.3	To determine the cognitive profile in neurologically asymptomatic HIV-infected patients before starting highly active antiretroviral therapy (HAART), and possible associated factors.
Characterization of HIV-Associated Neurocognitive Impairment in Middle-Aged and Older Persons With HIV in Lima, Peruf	Monica M Diaz; 2021	NCI	Prospective, Cross-sectional study; Lima, Peru	144	51.6	15.2	554^*^	The objective of this study was to analyze the rates and risk factors of NCI in middle-aged and older age PWH in Lima, Peru.
HIV disease dynamics, markers of inflammation and CNS injury during primary HIV infection and their relationship to cognitive performance	Longino; 2022	NCI	Prospective, longitudinal; Lima, Peru	87	27	0	510	The aim of this study was to examine the associations between NCI and CNS injury from HIV infection and ART
A Multinational Study of Neurological Performance in Antiretroviral Therapy-Naïve HIV-1-Infected Persons in Diverse Resource-Constrained Settings	Robertson; 2011	NCI	Prospective, cross-sectional; International	860	37	50	N/A	The objective of this study was o provide neurocognitive data for seven countries of limited resources.
Poor quality of life and incomplete self-reported adherence predict second-line ART virological failure in resource-limited settings	Torres; 2021	Mental Health	Prospective, Longitudinal study; International	500	39	51	​	To explore whether poorer quality of life predicts incomplete self-reported adherence to ART and early virologic failure in resource-limited settings.
Relationship between anxiety, depression and CD4+ T lymphocytes in patients with Human Immunodeficiency Virus (HIV) in a general hospital in Lima	Grados-Castro; 2023	Mental Health	Cross-sectional; Lima, Peru	144	41	28.50	596.36	To determine the relationship between anxiety, depression and CD4+ T lymphocyte count in a sample of PLWHIV.
HIV and antiretroviral treatment knowledge gaps and psychosocial burden among persons living with HIV in Lima, Peru	Navarro; 2021	Mental Health	Cross-sectional; Lima, Peru	171	36	30	N/A	This study aims to describe knowledge on HIV and antiretroviral (ARV) treatment and psychosocial factors among PWH in Lima, Per ´u, to explore characteristics associated to this knowledge, and determine its impact on sustained viral suppression.
High levels of mild to moderate depression among men who have sex with men and transgender women in Lima, Peru: implications for integrated depression and HIV care	Galea; 2022	Mental Health	Retrospective, cross-sectional; Lima, Peru	185	27	0	N/A	The aim of this study is to inform depression care integration in a community-based setting.
Risk Factors for Depression Among Middle-Aged to Older People Living With HIV in Lima, Peru	Failoc-Rojas; 2024	Mental Health	Retrospective Cross-sectional; Lima, Peru	139	51.7	15.3	553.5	The aim of this study was to identify depression risk factors among a group of middle-aged and older PWH in Lima, Peru.
Burden of Depression Among Improvished HIV-Positive Women in Peru	Wu; 2008	Mental Health	Observational cross-sectional analysis; Lima, Peru	78	N/A	100	N/A	The aim was to find the extent, risk factors, and experiences of depression among lower SES HIV positive women in Lima, Peru.
Mental Health Burden Among Impoverished HIV-Positive Patients in Peru	Shin; 2011	Mental Health	Longitudinal, qualitative analysis; Lima, Peru	95	N/A	N/A	N/A	The aim of this study was to understand factors contributing to poor emotional health and its impact among impoverished Peruvian HIV-infected individuals.
Metabolic profile and cardiovascular risk factors among Latin American HIV-infected patients receiving HAART	Cahn; 2010	CVD/Metabolic Syndrome	Prospective, longitudinal; Latin America	417	39.1	26.10	255	The objective of this study was to determine the prevalence of metabolic abnormalities (MA) and estimate the 10-year risk for cardiovascular disease (CVD) among Latin American HIV-infected patients receiving highly active anti-retroviral therapy (HAART).
Insulin resistance by homeostasis model assessment in HIV-infected patients on highly active antiretroviral therapy: cross-sectional study	Guillen; 2015	CVD/Metabolic Syndrome	Cross-sectional; Lima, Peru	219	38.4	32.90	N/A	The aim of this study was to determine the prevalence of insulin resistance (IR) in a cohort of human immunodeficiency virus (HIV)-infected patients on highly active antiretroviral therapy (HAART) and to investigate the potentially associated factors.
Prevalencia de factores de riesgo cardiovascular en una población peruana de pacientes con infección por el virus de inmunodeficiencia humana en terapia antiretroviral de gran actividad.	Rodriguez;2007	CVD/Metabolic Syndrome	Prospective,, cross-sectional; Lima, Peru	276	42	26.80	331	The objective of this study was to evaluate the prevalence of cardiovascular risk factors in a Peruvian cohort of patients with HIV in HAART
Elevada frecuencia de dislipidemia en pacientes infectados por VIH en un hospital público Peruano	Rondan; 2017	CVD/Metabolic Syndrome	Retrospective, cross-sectional; Lima, Peru	538	N/A	24	N/A	The objective of the study was to determine the frequency and characteristics of dyslipidemia in patients with HIV in highly active antiretroviral therapy.
Metabolic syndrome in HIV-infected patients receiving antiretroviral therapy in Latin America	Alvarez; 2010	CVD/Metabolic Syndrome;	Prospective, longitudinal; Latin America	4010	41.9	26.10	417	To evaluate the prevalence of and the associated factors for metabolic syndrome (MS) among Latin American HIV-infected patients receiving antiretroviral therapy (ART)
Metabolic and Cardiovascular Comorbidities Among Clinically Stable HIV Patients on Long-Term ARV Therapy in Five Ambulatory Clinics in Lima-Callao, Peru	Hidalgo; 2018	CVD/Metabolic Syndrome	Retrospective, cross-sectional; Lima-Callao, Peru	305	46	24	614.2	This study aimed to describe the most frequent cardiometabolic comorbidities found among ambulatory adults on ARV in Peru.
Concordancia entre las escalas de riesgo cardiovascular procam y framingham en varones que reciben tratamiento antirretroviral en un hospital nacional de Lima, Perú 2013	Pino; 2018	CVD/Metabolic Syndrome	Prospective, Cross-sectional; Lima, Peru	111	47	N/A	N/A	The aim of the study is to determine the concordance between the PROCAM (Prospective Cardiovascular Münster) and Framingham scales in patients receiving highly active antiretroviral therapy (HAART).
Assessment of regional body composition, physical function and sarcopenia among peruvian women aging with HIV: A cross-sectional study	Cabrera; 2023	Bone and Musculoskeletal Disorders	Prospective cross-sectional; Lima, Peru	316	52.4	100	591.5^*^	The objective of this study was to measure physical function, physical strength and sarcopenia among middle-aged and older Peruvian women with and without HIV.
Prevalence of and risk factors for vertebral fracture and low bone mineral density among Peruvian women aging with HIV	Cabrera; 2023	Bone and Musculoskeletal Disorders	Prospective cross-sectional; Lima, Peru	316	52.4	100	593.2	The objective of this study was to explore the prevalence of vertebral fracture (VF) and low bone mineral density (BMD) among women aging with HIV in Peru and identify risk factors for osteoporosis and fracture in this population.
Cancer in HIV-infected Persons from the Caribbean, Central and South America	Fink; 2011	NADM	Retrospective longitudinal; International	455	42^*^	14	92^*^	The objective of this study was to analyze cancers among HIV infected individuals in Latin America and the Caribbean.
Cancer in people living with HIV-AIDS at a referral hospital in Lima, Peru	Mendoza-Mori; 2021	NADM	Retrospective cross-sectional cohort study; Lima, Peru	276	42.2	20.60	112	The aim of this study was to describe and compare the demographic, clinical, and therapeutic characteristics of HIV patients who developed some cancer.

^*^Median reported.

The sample sizes ranged from 19 to 4,010 participants, with the largest studies representing international cohorts that included approximately 1,073 participants altogether from Peru. All studies enrolled adults aged 18 years or older (mean age range 27-56 years). Two studies did not report ages but specified that participants were adults. The proportion of female participants varied widely, ranging from 0% to 100%. We found no studies on frailty, disability, or renal disease in Peruvian PWH.

### Neurocognitive Impairment (NCI)

Five of the 23 articles described neurocognitive impairment (NCI) in PWH (9-12),^31^ which was commonly evaluated through comprehensive neuropsychological assessments that involve a battery of standardized tests. Supplementary Table 1 provides additional detail regarding the assessment instruments and outcome definitions used across studies evaluating neurocognitive impairment and mental health outcomes.

Two studies in our review^11,12^ assessed NCI using neurological examination and a complete neuropsychological evaluation conducted before and four months after ART initiation in PWH. Before starting ART, the majority of patients had low CD4 counts and exhibited significant cognitive impairment, particularly in executive function and psychomotor speed, with 95.2% failing at least one test. After ART initiation, improvements were noted in psychomotor speed and short-term visual memory, suggesting that some aspects of NCI may be reversible and that cognitive decline may begin early in HIV infection.

Another study of 87 young men who have sex with men (MSM) and transgender women with acute HIV infection conducted longitudinal neuropsychological testing and biomarker sampling before and after ART initiation. Biomarkers of central nervous system (CNS) inflammation, immune activation, and neuronal injury peaked early but declined after ART, without significant correlations with cognitive performance over 192 weeks. The neuropsychological battery administered for this study included Timed Gait, Grooved Pegboard, and finger tapping, Color Trails 1 and 2, Stroop Color Word Interference Test, Animal Fluency, and the Hopkins Verbal Learning Test-Revised. Neurocognitive performance remained within the normative range throughout, with only modest, clinically insignificant improvements. These findings suggest that early CNS injury markers do not predict long-term cognitive outcomes in this cohort initiating ART during acute HIV infection.^13^

Another study of Peruvian PWH found that nearly 30% had global NCI, and all were classified as asymptomatic or mild neurocognitive impairment. Assessments included a range of neuropsychological tests such as the Color Trail Test, Grooved Pegboard, Hopkins Verbal Learning Test - Revised, and Wechsler Adult Intelligence Scale - 3 Digit Span. The most common impairments were in attention and working memory (70% of participants), followed by information processing speed (18%). No HIV-related factors predicted NCI, but a history of treated syphilis or tuberculosis was associated with an increased risk.^10^

An international study including Peruvian PWH found that Peruvian participants had the lowest rates across various neurological performance with 8% of these participants having neurological dysfunction. This finding may be a result of la higher educational level compared with those from other countries (median educational level 12.5 years). The study highlighted education as a key factor influencing performance across a wide range of neuropsychological assessments, including Hopkins Verbal Learning Test-Revised, WAIS-III Digit Symbol, Grooved Pegboard, and Color Trails 1 & 2 tests.^31^

Across studies, the cognitive domains most affected by HIV varied, though deficits in executive function, psychomotor speed, and working memory/attention were consistently reported. Differences in domains were also observed between sexes. The initiation of ART was found to improve performance in some cognitive domains. These findings highlight the varied NCI impairment detected across studies and the differing batteries of neuropsychological tests applied that may introduce variability across studies.

### Mental Health

Seven studies reported prevalence of depression and its manifestations.^21-26,30^ Among these studies, the prevalence of depression among PWH varied widely. In one study, anxiety and depression were reported in 34% and 16.7% of patients on ART, respectively, using the Hospital Anxiety and Depression Scale among ART-treated PWH.^22^ Conversely, in another study, nearly half (48%) of PWH screened positive for some grade of depression using the Mental Health Inventory-5, with moderate depression and shorter duration of ART linked to poor knowledge of HIV and ART. The observed differences in prevalence of depression may in part be due to differences in depression screening instruments utilized across studies or differences in the populations that were surveyed (hospital-based vs. community-based), but high rates of mental health symptoms among PWH were still demonstrated.

In another study using the Patient Health Questionnaire-9 (PHQ-9), about 25% of participants were found to be depressed. Being female, current smoking status and having had a prior opportunistic infection were risk factors for depression. Additionally, adherence to ART was associated with 50% less depression.^26^ This highlights the importance of promoting ART adherence, particularly in PWH who screen positive for depressive symptoms. In another study, 42% of participants were found to be depressed using the PHQ-9, 81% of whom had mild to moderate depression.^21^ Using the Hospital Anxiety and Depression scale, one study found that more advanced AIDS stage and chronic comorbidities were associated with significantly higher levels of both anxiety and depression. This highlights how chronic comorbidities of HIV, such as hypertension, diabetes, syphilis, hypothyroidism, and cirrhosis, can exacerbate mental health burden among Peruvian PWH.^22^ Interestingly, in another study, lower health-related quality of life scores, particularly in cognitive functioning, pain, and mental health domains, were associated with higher rates of early virologic failure and worse ART adherence.^24^ Additionally, a qualitative study found that chronic stress, economic hardship, fragmented family relationships, and substance use significantly contributed to the mental health burden among PWH.^30^

These studies highlight the importance of addressing the mental health burden placed on PWH, particularly those facing socioeconomic challenges, which may lead to lower ART adherence and adverse health outcomes. Efforts to improve HIV knowledge, ART adherence, and provide mental health support are crucial for improving the well-being of PWH in Peru.

### Dyslipidemias and Metabolic Syndrome

Seven total studies were included that discussed dyslipidemia, cardiovascular disease, or metabolic syndrome. Metabolic syndrome is defined as a condition that elevates the risk of cardiovascular diseases, such as coronary heart disease, diabetes or stroke. Some conditions include increased waist circumference, hypertension, hyperglycemia, high blood triglycerides and low HDL cholesterol. Mean ages across studies ranged from 38 to 47 years. The prevalence of cardiovascular disease among Peruvian PWH was found to be relatively low but was closely associated with metabolic comorbidities, such as dyslipidemia.

One study found that 34.2 % of PWH on ART showed insulin resistance determined by the insulin resistance homeostasis model assessment mathematical model.^14^ Additionally, the authors found that 26.9% of PWH had metabolic syndrome. Age older than 46 years and higher body mass index were associated with insulin resistance.^14^ Furthermore, in another study, metabolic syndrome was found to affect 20.2% of the total cohort across all ages, with older individuals exhibiting a higher risk of developing metabolic syndrome.^27^

In a cohort of 111 men living with HIV on ART (median age 47 years), 76.6% were diagnosed with dyslipidemia. Specifically, 81.2% had total cholesterol levels ≥200 mg/dL, 69.4% had triglyceride levels ≥200 mg/dL, and 44.7% had LDL cholesterol levels ≥160 mg/dL.^16^ The presence of high cardiovascular risk was generally, low with only 5.4% of participants classified as high-risk, according to the Framingham Risk Score (FRS), and 3.6% used the PROCAM risk tool.^16^ Similarly, in another study, dyslipidemia was found in 74.5% of participants using criteria defined by the NCEP-ATP III. The use of protease inhibitors in ART regimens and being older than 40 were associated with dyslipidemia.^18^

In a study of PWH from seven Latin American countries, dyslipidemia was also highly prevalent, with an observed intermediate 10-year risk for developing CVD.^28^ Longer exposure to ART was significantly associated with an increased risk of dyslipidemia, type 2 diabetes, and metabolic syndrome. Additionally, each month of HAART exposure increased the risk of developing CVD in 10 years, as seen by increasing the FRS by 0.09 per month of exposure. CVD risk by 0.09 according to the FRS. Male patients were found to have a higher CVD risk compared to females in this cohort.^28^ The analysis of older age groups revealed that hypertension was more prevalent among individuals aged ≥50 years, with a significant difference between those with and without hypertension (18.9% vs. 3.1%; p < 0.001).^15^ Hypertension was also more strongly associated with older age. These studies all demonstrate high rates of dyslipidemia and metabolic syndrome highlighting the need for early identification of dyslipidemia in order to begin appropriate cholesterol treatment and improve diet and lifestyle modification to reduce cardiovascular risk.

### Bone and Musculoskeletal Disorders

Two studies were included that investigated bone and musculoskeletal disorders among Peruvian PWH.^19,20^ Both studies were conducted in the same population of Peruvian women living with HIV and explored differences in bone and musculoskeletal disorders between women living with and without HIV. One study reported that among postmenopausal women, osteoporosis prevalence was comparable between women with and without HIV (26.3% vs. 25.9%).^19^ However, vertebral fractures were twice as common in women living with HIV compared to women without HIV (12.5% vs. 6.2%).^19^ This finding suggests that women living with HIV have osteoporosis rates comparable to those of older women without HIV. Although national guidelines recommend initiating osteoporosis treatment based on dual X-ray absorptiometry (DXA) and Fracture Risk assessment (FRAX) scores, a substantial number of individuals living with HIV met these criteria, yet none were receiving any treatment. Further analysis using propensity score matching demonstrated that women with HIV had significantly lower femoral neck and total hip bone mineral density, suggesting a greater risk for fracture and bone loss progression.^19^

Another study examining body composition and physical performance found that women with HIV had significantly lower Short Physical Performance Battery scores, indicating impaired physical function. Although overall sarcopenia rates were similar between groups, severe sarcopenia was more frequent among women with HIV, and higher trunk fat mass was independently associated with poorer physical performance.^20^ These two studies highlight the need to improve screening for and treatment of osteoporosis and sarcopenia in women with HIV in Peru.

### Non-AIDS Defining Malignancies

We found only two manuscripts that explored non-AIDS defining malignancies (NADM) diagnoses among Peruvian PWH.^9,29^ One study retrospectively analyzed hospitalized PWH in a hospital in Lima, Peru^9^ and found 276 malignancies diagnosed between 2000 and 2018.^9^ Of these, 19.2% were NADM. PWH with NADM had a median age of 42.2 and a male-to-female ratio of 3:2. Hodgkin’s lymphoma was the most prevalent diagnosis, making up 22.6% (12/53) of all NADM, followed by cervical cancer and skin cancer making up 17% (9/53) and 13.2% (7/53) of all NADM, respectively.^9^

Another study including PWH from the Caribbean, Central and South America, including Lima, Peru, reported 406 malignancies from 1996 to 2008, with 20% classified as NADMs. Among those with NADMs, most were male at birth, the median age was 41, and 74% were diagnosed more than a year after their HIV diagnosis. In the Peruvian cohort, 42 malignancies were reported, 21% of which were NADMs, with cervical cancer being the most frequently diagnosed.^29^ These few studies highlight the need for more research on NADM which are known to be more prevalent among PWH.

## Discussion

Our review found that NCDs, including CVD, MSK disorders, NCI and mental health conditions, are common among PWH in Peru. NCI prevalence ranged between 28.5-47.6%,^10,12^ and depression prevalence was high (range 16.7-68%).^22,25^ Dyslipidemia was seen in up to 76.6%,^16^ and metabolic syndrome was also common among PWH (ranging between 20.2-26.9%).^14,27^ Few studies examined bone and musculoskeletal disorders, but the few that did found that appropriate treatment for osteoporosis and sarcopenia among PWH is not currently being implemented in Peru.^19^ This review also highlights that of the NADM described, Hodgkin’s lymphoma and cervical cancer were the most common. Despite the high burden of these NCDs, they are commonly underrecognized in both clinical care and national public health strategies. Despite advancements in HIV treatment and care in Peru, recognition, including screening, diagnosis and management of chronic comorbidities of aging in PWH remains limited.^32^ While the National HIV Program has successfully increased access to ART and improved viral suppression rates, there is insufficient attention on the long-term health complications associated with aging with HIV and lifetime ART use.

One of the major gaps in HIV care in Peru is the lack of routine screening and management of chronic comorbidities in PWH, particularly as they age. Current healthcare models primarily focus on HIV suppression through ART but do not adequately address the broader spectrum of health issues that affect long-term outcomes.^33^ Research from other LAC countries has demonstrated that older PWH are at higher risk for multimorbidity, yet similar data in Peru remain scarce.^5^ Additionally, absolute CD4 count at diagnosis and treatment initiation remains an important factor influencing comorbidity risk, as lower CD4 counts have been associated with higher rates of inflammation-driven chronic diseases. However, Peruvian studies have yet to comprehensively examine the impact of immune dysregulation on long-term health outcomes in PWH.

Another critical aspect is the role of ART in accelerating or mitigating chronic disease progression.^33^ While ART has been instrumental in reducing AIDS-related mortality, its long-term metabolic effects, including increased risk for hypertension, dyslipidemia, and insulin resistance, require closer monitoring. In Peru, there is limited research assessing the impact of specific ART regimens on chronic disease development, making it difficult to establish targeted clinical guidelines for aging PWH. Moreover, polypharmacy among older PWH presents significant challenges, not only due to potential drug interactions between ART and medications for chronic conditions which may affect treatment adherence and health outcomes, but also because it is associated with falls, functional impairment, prolonged hospital stays, readmissions, and increased mortality.^34^

Recognizing chronic comorbidities in PWH is necessary to improve patient care and ensure long-term health outcomes improve over time. Although this review was motivated by the growing population of people aging with HIV globally, the available literature in Peru largely reflects younger and middle-aged populations. The mean age across studies ranged from approximately 27 to 56 years, and few studies reported comorbidity outcomes stratified by age. As a result, the evidence summarized here likely reflects early patterns of chronic comorbidities rather than the full burden of aging-related multimorbidity among older adults living with HIV in Peru. An additional challenge in interpreting the literature is the heterogeneity in measurement approaches across studies. For example, studies assessing depression and neurocognitive impairment used different screening tools and diagnostic thresholds. This variability limits direct comparisons of prevalence estimates across studies and highlights the need for methodological standardization in future studies.

A shift is needed in Peruvian HIV care, moving from viral suppression as the primary endpoint to a more comprehensive approach that integrates chronic disease management into routine HIV care. Routine screening for NCDs, early interventions for at-risk individuals and interdisciplinary care models are necessary to address the growing burden of chronic comorbidities. Moreover, research efforts should focus on generating local data on the prevalence, risk factors, and outcomes of NCDs in PWH to inform evidence-based policies and clinical guidelines.

Our review highlights the urgent need to shift the focus toward chronic comorbidities in Peru’s HIV response. As PWH live longer, the healthcare system must adapt to the changing epidemiological landscape by prioritizing chronic disease prevention and management. Failure to address this issue may compromise the gains achieved in HIV care and negatively impact the quality of life for PWH in Peru.

Among the 23 studies included in this review, most did not disaggregate findings by age, limiting our understanding of age-related disease burden. This is consistent with trends across Latin America and the US. The studies included in this review addressed a range of comorbidities, with the most common being CVD, mental health conditions, NCI, and metabolic disorders such as diabetes, hypertension, and dyslipidemia. However, there were no studies that assessed frailty nor disability in Peru, which are syndromes increasingly relevant to PWH.

Some limitations of the studies include estimates that varied widely which may have been due to various assessment tools utilized, making it difficult to compare comorbidity rates. Variations in tools, definitions, and diagnostic criteria can lead to inconsistent and non-equivalent measurements. This highlights the need for a standardized approach to evaluating NCDs across the country suited to the Peruvian context. Secondly, most of the studies included in this review were cross-sectional. A small number of studies used a longitudinal design with repeated follow-up assessments, primarily those studies that assessed neurocognitive impairment and mental health outcomes. This limits our understanding of how major comorbidities progress or emerge over time, which is particularly important as people living with HIV age and become more susceptible to developing additional chronic conditions that may not be apparent in cross-sectional studies. Longitudinal studies are needed to assess incident comorbidities over time in Peru among PWH. Next, there was limited representation of older adults living with HIV. Most studies included relatively young populations and did not stratify outcomes by age. This limits the ability to directly assess the burden of aging-related comorbidities among individuals aged ≥50 years. Future research in Peru should prioritize age-stratified analyses and longitudinal studies of older adults living with HIV to better characterize multimorbidity and functional outcomes with aging. Next, our search strategy was limited to PubMed and LILACS. Although these databases provide coverage of biomedical literature and regional publications from Latin America, it is possible that relevant studies indexed exclusively in other databases (e.g., Embase, Scopus, or Web of Science) were not captured. However, the inclusion of LILACS helps mitigate this limitation by identifying regional journals that are often not indexed in other major databases. Next, the included studies also used a variety of instruments and definitions to assess comorbidities such as depression and neurocognitive impairment. Because this scoping review aimed to describe the reported comorbidities rather than conduct a detailed methodological comparison of measurement approaches, we did not systematically extract instrument-level information. Lastly, few studies focused on certain chronic comorbidities, restricting our ability to make comprehensive conclusions about their prevalence and impact among PWH in Peru.

## Conclusions

The growing burden of NCDs among PWH in Peru reflects an increase in NCDs among PWH worldwide. Few studies exist on particular comorbidities, such as NAMD and bone and musculoskeletal disease for Peruvians, highlighting an area of research that should be expanded. Despite widespread ART coverage and improvements in HIV survival, national programs have not yet adapted to include NCD screening and management within the HIV national care program. Integration of HIV and chronic disease management, particularly for aging adults, remains limited. Our findings highlight the need for an integrated, multidisciplinary response that includes routine screening, early intervention, and longitudinal care for neurocognitive, mental health, cardiovascular, metabolic, and musculoskeletal comorbidities. Strengthening the National STD/HIV Program to include these priorities is essential for ensuring the long-term health and quality of life of PWH in Peru.

## Supplemental Material

Supplemental material - A Review of Chronic Comorbidities in People Living With HIV in Peru Supplemental material for A Review of Chronic Comorbidities in People Living With HIV in Peru by Marleny Nolasco, Evelyn Hsieh, Patricia J. Garcia, Monica M. Diaz in Journal of the International Association of Providers of AIDS Care (JIAPAC)

## Data Availability

The authors can make the data available with an appropriate request to the corresponding author.*
