# Supplementary material for: A Review of Chronic Comorbidities in People Living With HIV in Peru
Source: J Int Assoc Provid AIDS Care. 2026 Jul 17;25:23259582261470497. doi: 10.1177/23259582261470497 (PMC13379654; doi:10.1177/23259582261470497)
Supplement: Supplemental material - A Review of Chronic Comorbidities in People Living With HIV in Peru [file sj-pdf-1-jia-10.1177_23259582261470497.pdf]

Supplementary Table 1. Neurocognitive and Mental Health Assessment Methods Across Included Studies

Title	Author	Comorbidity	Type Of Study	N	Prevalence	Neurocognitive Assessment	Scoring
Cognitive impairment and antiretroviral treatment in a Peruvian population of patients with human immunodeficiency virus	Guevara-Silva; 2014	NCI	Longitudinal study; Lima, Peru	19	1-13 people failed each test pre-ART. 5-15 people failed 3 out of 6 tests post-ART.	Neuropsychological battery compromised of 6 tests:<br>• Trail Making Test A & B;<br>• Digit symbol coding;<br>• Rey Auditory Verbal Learning Test;<br>• Digit Span; and<br>• Complex Figure Test	Assessed changes in scores across all tests to identify changes in different cognitive domains over time.
Cognitive profile in human immunodeficiency virus-infected neurologically asymptomatic patients	Guevara-Silva; 2013	NCI	Cross-sectional study; Lima, Peru	21	47.60%	Neuropsychological battery compromised of 6 tests:<br>• Trail Making Test A & B;<br>• Digit symbol coding;<br>• Rey Auditory Verbal Learning Test;<br>• Digit Span; and<br>• Complex Figure Test	Cognitive impairment was defined as failure on three or more of the administered tests.
Characterization of HIV-Associated Neurocognitive Impairment in Middle-Aged and Older Persons With HIV in Lima, Peruf	Monica M Diaz; 2021	NCI	Cross-sectional study; Lima, Peru	144	28.60%	Neuropsychological test battery assessing seven domains:<br>• Abstraction and executive function: Color Trails Test 2;<br>• Motor performance: Grooved Pegboard (dominant hand) and Grooved Pegboard (non-dominant hand);<br>• Memory (learning and recall): Hopkins Verbal Learning Test Revised (HVLT-R)-Total Learning, HVLT-R Delayed Recall , and Benson Figure Recall;<br>• Attention and working memory: Weschler Adult Intelligence Scale (WAIS)-3 Digit Span;<br>• Verbal and language fluency: semantic/category fluency (Animal Naming) and letter fluency (PMR);<br>• Speed of information	A Global Deficit Score (GDS), a measure of global cognitive impairment was computed. The GDS summarizes the number and severity of neurocognitive deficits across the entire test battery. A GDS cutoff of ≥0.50 was used to determine global NCI.

						processing: Color Trails Test 1; and<br>• Visuospatial orientation: Benson Figure immediate copy	
HIV disease dynamics, markers of inflammation and CNS injury during primary HIV infection and their relationship to cognitive performance	Longino; 2022	NCI	Longitudinal; Lima, Peru	87	Not reported.	The neuropsychological battery included tests the following tests:<br>• Gross Motor: Timed Gait;<br>• Fine Motor Speed: Grooved Pegboard and Finger Tapping (dominant and nondominant hands);<br>• Psychomotor/Executive Function: Color Trails 1 and 2, Stroop Color Word Interference Test;<br>• Language: Animal Fluency,<br>•Verbal Learning and Memory: Hopkins Verbal Learning Test-Revised (HVLT-R).	Age- and education-adjusted z scores using local normative data were averaged across tests to derive an overall NP score.
A Multinational Study of Neurological Performance in Antiretroviral Therapy-Naïve HIV-1-Infected Persons in Diverse Resource-Constrained Settings	Robertson; 2011	NCI	Prospective cross-sectional study; International	860	6% (MND)<br>n = 4 (HAD)	The tests administered were:<br>• Timed Gait, Grooved Pegboard (dominant and nondominant hands);<br>• Finger Tapping (dominant and nondominant hands); and<br>• Semantic Verbal Fluency	Each patient was used as their own control, and comparison of the neuropsychological treatment response over time was the main outcome. The neurological exam summary assessment of diffuse CNS disease related to HIV of subclinical/equivocal was used coded for MND, and the mild, moderate or severe ratings were coded as HAD.
Poor quality of life and incomplete self-reported adherence predict second-line ART virological failure in resource-	Torres; 2021	Mental Health	Longitudinal; International	500	Not reported.	Quality of life was measured using the ACTG SF-21 which covers the following eight domains:<br>General Health Perceptions (GHP), Physical Functioning (PF), Role Functioning (RF),	A standardized score ranging from 0 to 100 was calculated for each domain with 100

limited settings						Social Functioning (SF), Cognitive Functioning (CF), Pain (P), Mental Health (MH), and Energy/Fatigue (E/F)	representing best QoL.
Relationship between anxiety, depression and CD4+ T lymphocytes in patients with Human Immunodeficiency Virus (HIV) in a general hospital in Lima	Grados-Castro; 2023	Mental Health	Cross-sectional; Lima, Peru	144	34% had some level of anxiety. 16.7% had some level of depression.	Hospital Anxiety and Depression Scale (HADS)	Anxiety was defined using the following scores: no anxiety (0–8), mild (9–10), moderate (11–13), severe (≥ 14). Depression was defined using the following scores: no anxiety (0–8), mild (9–10), moderate (11–13), severe (≥ 14)
HIV and antiretroviral treatment knowledge gaps and psychosocial burden among persons living with HIV in Lima, Peru	Navarro; 2021	Mental Health	Cross-sectional study; Lima, Peru	171	48%	Mental Health Inventory-5 (MHI5) scale	Does not detail cutoff scores or definitions. Based on internet search a score below 60 = positive screening for probable distress
High levels of mild to moderate depression among men who have sex with men and transgender women in Lima, Peru: implications for integrated depression and HIV care	Galea; 2022	Mental Health	Retrospective cross-sectional analysis; Lima, Peru	185	42% had some level of depression.	Patient Health Questionnaire-9 (PHQ)-9	the following standard PHQ-9 cut-off scores were used: minimal/no depression (PHQ-9 = 0–4); mild depression (PHQ-9 = 5–9); moderate depression (PHQ-9 = 10–14); moderately-severe depression (PHQ-9 = 15–19); and severe depression (PHQ-9 = 20–27)
Risk Factors for Depression Among Middle-Aged to Older People Living	Failoc-Rojas; 2024	Mental Health	Qualitative Study; Lima, Peru	139	25% had some level of depression.	Patient Health Questionnaire (PHQ)-9	The standard cutoff score to identify any depression (mild, moderate, or severe) is 5 or

With HIV in Lima, Peru							above.
Burden of Depression Among Improvised HIV-Positive Women in Peru	Wu; 2008	Mental Health	Observational Cross-sectional analysis; Lima, Peru	78	68%	Hopkins Symptom Checklist-15 to assess depression	Questions were scored on a severity scale from 1 to 4, with a higher score indicating more depressive symptoms with the conventional cutoff score of 1.75
Mental Health Burden Among Impoverished HIV-Positive Patients in Peru	Shin; 2011	Mental Health	Longitudinal qualitative analysis; Lima, Peru	95	N/A	N/A	N/A
